# Proinflammatory cytokines interleukin-18 and interleukin-6 mediate anorexia induction by trichothecene deoxynivalenol and its congeners

**DOI:** 10.3389/fvets.2024.1521424

**Published:** 2024-12-03

**Authors:** Chuang Zhou, Zihui Qin, Huayue Zhang, Huiping Xiao, Hua Zhang

**Affiliations:** ^1^Jiangsu Vocational College of Agriculture and Forestry, Jurong, China; ^2^College of Veterinary Medicine, Gansu Agricultural University, Lanzhou, China; ^3^School of Food and Biological Engineering, Engineering Research Center of Bio-process, Ministry of Education, Hefei University of Technology, Hefei, China; ^4^Food Laboratory of Zhongyuan, Luohe, China

**Keywords:** deoxynivalenol, trichothecene, anorexia, interleukin-18, interleukin-6

## Abstract

As the common foodborne mycotoxins with the highest pollution rate, deoxynivalenol (DON, also named “vomitoxin”) can harm the health of humans and animals by causing anorectic response. It has four congeners: 3-acetyldeoxynivalenol (3-ADON), 15-acetyldeoxynivalenol (15-ADON), nivalenol (NIV), and fusarenon X (FX). These five mycotoxins have been associated with the detrimental effect on food intake. However, its underlying mechanism of anorexia remains unclear. The goal of this research was to compare the anorectic responses to these five mycotoxins and relate these effects to proinflammatory cytokines interleukin-18 (IL-18) and interleukin-6 (IL-6) following intraperitoneal (IP) and oral exposure to a common dose at 2.5 mg/kg BW in mice. Plasma IL-18 and IL-6 were elevated within 1–2 h and returned to basal levels at 6 h after exposure to DON, 3-ADON and 15-ADON. FX promoted IL-18 expression at 6 h. Whereas, FX only promoted IL-6 at 6 h. When NIV was injected intraperitoneally, IL-18 started to rise at 1 h and peaked at 6 h. Whereas, NIV only promoted IL-18 at 2 h following oral exposure. IP exposure to NIV induced an increase in IL-6 that occurred only at 2 h. No effect on IL-6 when exposed orally to NIV. In conclusion, the data indicate that IL-18 and IL-6 play critical roles in anorectic response induced by DON and its four congeners 3-ADON, 15-ADON, NIV, FX.

## 1 Introduction

Mycotoxins are harmful products produced by fungi widely contaminating cereals and cereals products (1, 2). DON is one of the most frequently contaminated mycotoxins and belongs to the type B trichothecene. In addition to DON, the type B trichothecenes also include 3-acetyldeoxynivalenol (3-ADON), 15-acetyldeoxynivalenol (15-ADON), nivalenol (NIV), and fusarenon X (FX). Both 3-ADON and 15-ADON are the acetylated form of DON, while FX is the acetylated form of NIV (3–5). 3-ADON, 15-ADON and FX can also be converted to their parent forms *in vivo*. Several mycotoxin contamination surveys (6–8) have shown that the coexistence of these trichothecenes is very common. These mycotoxins can contaminate a wide range of cereals including maize, wheat, barley and oats. Results from 8 years of field surveys in wheat cultivated in the Netherlands showed that DON was detected in 54% of the samples (>50 μg/kg) ranging from 19 to 92% depending on the year, and the presence of 3-ADON, 15-ADON and NIV were up to 8% in some years (9). Ingestion of these contaminated foods and feeds by humans and animals can lead to poisoning. Type B trichothecenes have a tetracyclic 12,13-epoxy monosporin backbone and are able to bind to eukaryotic ribosomes, causing strong cytotoxicity to eukaryotic cells, as well as binding and disrupting protein synthesis through interaction with peptidyl transferase centers (4, 10). Acute and chronic exposure can also lead to anorexia, vomiting, immunotoxicity, and genotoxicity (11, 12). Ingestion of feed contaminated with these mycotoxins by farm animals can lead to reduced intake and slower weight gain, resulting in significant economic damage (4, 12, 13). An incident of acute DON poisoning in 2019 (14) had symptoms that included mainly vomiting, nausea, and abdominal pain. In addition, prolonged intake of DON-contaminated food can lead to growth retardation in children, and anorexia is an essential factor contributing to growth retardation (15–18). Given the harmful effects of anorexia caused by DON and its congeners on humans and animals, it is necessary to investigate the specific mechanisms by which these mycotoxins cause anorexia.

Appetite is the foundation for the body to obtain food and maintain normal metabolism (19). Appetite regulation involves many aspects, including genetic, physiological, and environmental factors. The hypothalamus has long been recognized as a critical site of appetite regulation, responding to both peripheral and centrally generated orexigenic and anorexic signals (20, 21). As the most important part of appetite regulation in the hypothalamic nucleus, the arcuate nucleus (ARC) has two neurons with different functions, namely neuropeptide Y (NPY)/agouti-related peptide (AgRP) that promotes appetite and pro-opiomelanocortin (POMC) that suppresses appetite (22–24). The intestinal tract is another key hub for appetite regulation, with the secretion of intestinal satiety hormones that link signals in the gut to a range of physiological activities via the brain-gut axis (25). DON-induced anorexia can indirectly affect appetite by inducing a significant release of peripheral satiety hormones, in addition to upregulating central anorexigenic molecules such as POMC and melanocortin 4 receptor (MC4R) (26, 27). Our previous findings (25, 26, 28) suggest that DON and its four congeners 3-ADON, 15-ADON, NIV, FX induce anorexia in mice by regulating intestinal hormones including substance P (SP), glucagon-like peptide-17-36 amide (GLP-1), cholecystokinin (CCK). Moreover, inflammation also has been reported to be involved in the regulation of appetite, and the pro-inflammatory cytokines seems to play a critical roles in it (29, 30).

Inflammation is the body’s defensive response to various stimuli. However, dysregulated inflammation can disrupt the homeostasis of the body, resulting in a variety of acute and chronic diseases (31, 32). DON-induced inflammation has been reported in many species, including humans, mice, rats, and pigs (15, 33–36). The expression and release of various pro-inflammatory cytokines, including IL-6, IL-1β, TNF-*α*, and IL-18 were observed after DON exposure. Congeners of DON have also been shown to promote the pro-inflammatory cytokines (37–41). DON can promote IL-18 and IL-6 in the hypothalamus and intestine, which are closely related to the regulation of appetite (27, 42). Moreover, IL-18 and IL-6 are also considered to be involved in the regulation of appetite (43, 44). IL-18 is thought to be associated with poor appetite in acutely ill patients, while IL-6 is thought to be involved in the regulation of appetite after acute exercise in humans. Our previous findings (45) suggest a link between the anorexic induced by oral exposure to DON and the release of TNF-*α* and IL-1β. Both IL-1β and TNF-α receptor antagonists could weak the DON-induced anorexia. Furthermore, anorectic responses to DON’s four congeners 3-ADON, 15-ADON, NIV, FX correspond to secretion of TNF-α and IL-1β following both IP and oral exposure. However, whether IL-18 and IL-6 are involved in the anorexia induced by DON and its congeners is not clear.

In order to investigate the potential roles of pro-inflammatory cytokines IL-18 and IL-6 in anorexia induction, mice were exposed with a common anorexigenic dose of 2.5 mg/kg BW DON, 3-ADON, 15-ADON, NIV and FX by two methods (IP vs. oral) using a previously established mouse anorexia model (46). The results presented herein indicate that following both IP and oral treatment, DON and its four congeners 3-ADON, 15-ADON, NIV, FX evoked significant anorectic responses; DON and its four congeners 3-ADON, 15-ADON, FX, NIV significantly upregulated plasma IL-18 and IL-6 in mice; Anorectic response induction by DON and its four congeners 3-ADON, 15-ADON, NIV, FX correspond to release of plasma IL-18 and IL-6 in mice.

## 2 Materials and methods

### 2.1 Toxins

Both DON and 3-ADON was produced, identified by nuclear magnetic resonance (NMR) and supplied by Dr. Tony Durst (University of Ottawa, Canada). 15-ADON was isolated from culture, identified by high performance liquid chromatography (HPLC) and provided by Dr. James J. Pestka (Michigan State University, USA) (41). FX and NIV were purified from cultures and supplied by Dr. Yoshiko Sugita-Konishi (Azabu University, Japan). The purity of the five trichothecene toxins used in the experiments was analyzed using LC–MS and elemental analysis to ensure that the toxin concentrations were > 98%. Five toxins were all dissolved in sterile PBS.

### 2.2 Animals

11–12 week-old female B6C3F1 mice were obtained from Vital River Laboratory Animal (Beijing, China) and housed individually in polycarbonate cages. Room temperature and humidity maintained at 19–23°C and 30–70%, respectively. Mice were fed and watered *ad libitum* and given a 12 h light/dark cycle. High fat diet (45 kcal% fat diets, Jiangsu Medicine Company, Yangzhou, China) put in 2 inch high glass jars was employed for feeding bioassay and sifted aspen chips used for bedding. No deaths were eliminated during the whole period. All animal handling in the experiments followed the guidelines of the Nanjing Agricultural University Institutional Animal Care and Use Committee (Certification No: SYXK (Su) 2011–0036).

### 2.3 Experimental design

**Figure 1:**
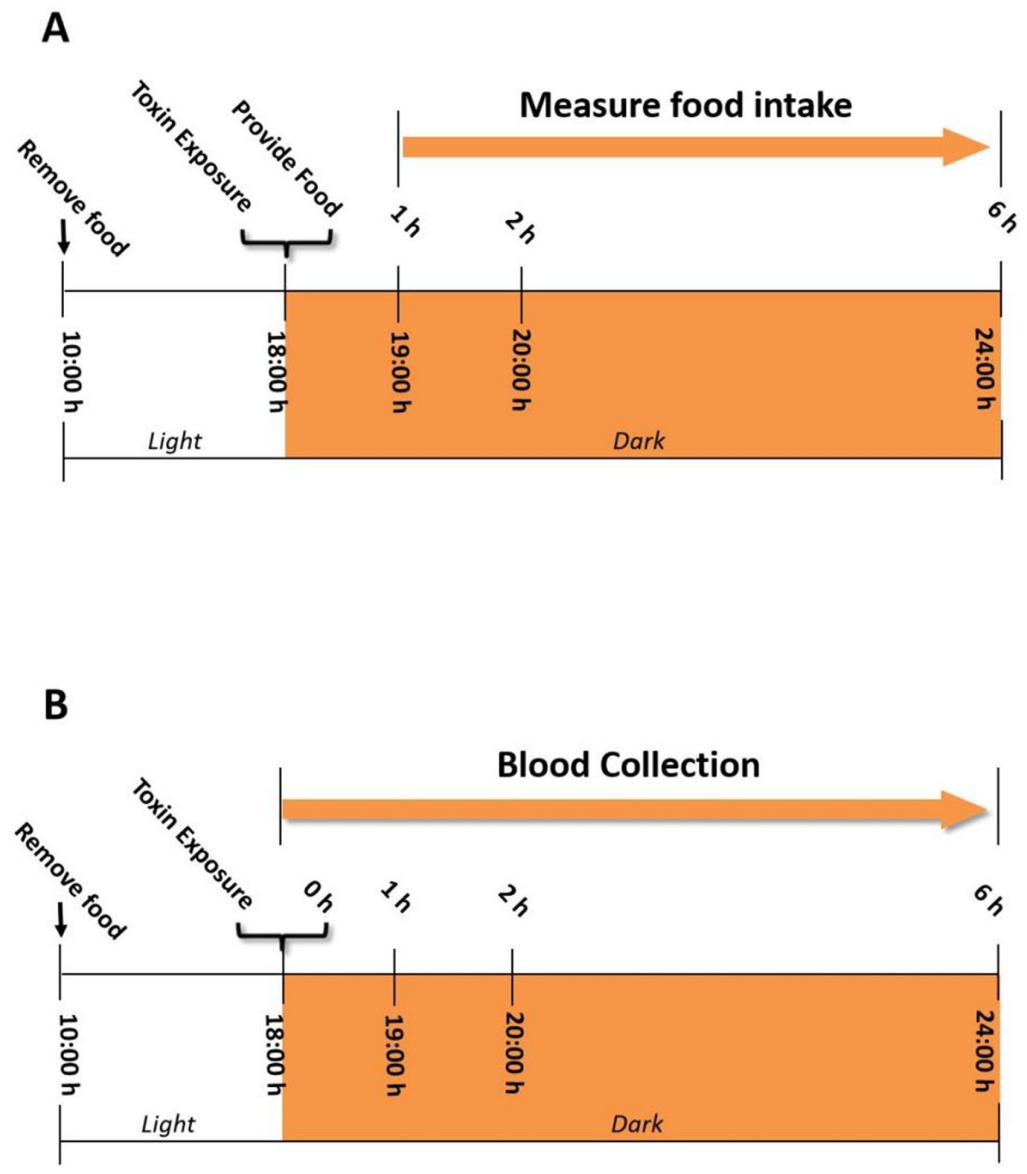
**(A)** Experimental design for anorectic response bioassay in the mouse. Mice were given IP injection or oral gavage of DON and its four congeners 3-ADON, 15-ADON, FX, NIV immediately before the dark feeding cycle. Food intake was measured at 1, 2, and 6 h post administration. **(B)** Experimental design for IL-18 and IL-6 induction by DON and its four congeners 3-ADON, 15-ADON, FX, NIV in the mouse plasma. Plasma was analyzed for IL-18 and IL-6 at 0, 1, 2, and 6 h post administration.

Figure 1 summarizes the general experimental design based on preliminary experiments in this study. All handling procedures are consistent and fast to minimize stress on the mouse. The study of reducing the number of animals requires a flushing period of at least 1 week after the cessation of the IP mouse experiment treated with a separate toxin, and then randomized for subsequent oral challenge studies on the same toxin. The effectiveness of this method is based on our previous research (46), which showed that the response to DON was the same in mice given a 1-week flushing cycle, without significant DON attenuation or enhanced anorectic response.

#### 2.3.1 Experiment 1: anorectic response of five trichothecene toxins by IP and oral treatment

The experimental design is based on the previously established mouse anorexia model (46). Briefly, all mice were acclimatized for 1 week prior to the start of the experiment (Figure 1A). Randomly group mice based on their body weight the day before the experiment (*n* = 6). Fasting of mice from 10:00–18:00 on the day of the experiment but not water. Mice were given 100 μL of PBS containing 2.5 mg/kg BW of toxin by gavage and intraperitoneal injection at 18:00. The control group was given 100 μL of PBS. Mice were then immediately offered food pellets (approximately 7 g) and food intake was measured at 1 h, 2 h and 6 h.

#### 2.3.2 Experiment 2: effect of DON and its congeners on IL-18 and IL-6

The exposure pattern and dose of toxin in mice are the same as in experiment 1 except not providing food pellets (Figure 1B). Mice were anesthetized using sodium pentobarbital as per ethical considerations and sacrificed 0, 1, 2, 6 h after exposure to the toxin. Blood was collected using an EDTA-containing blood collection tube, followed by centrifugation for 10 min (3,500 × g, 4°C) to collect plasma. Plasma IL-18 and IL-6 levels were measured by enzyme-linked immunoassay kits (R & D systems).

### 2.4 Statistics

SigmaPlot (Jandel Scientific; San Rafael, CA) was used to conduct data calculation. All the data are expressed as mean ± SEM, with *p*-values less than 0.05 to be considered as significant differences. A two-way repeated ANOVA with one factor using Holm-Sidak Method was used to calculate significant differences in food consumption. Besides, the kinetics of IL-18 and IL-6 concentrations in the mouse plasma was analyzed by a 2-way ANOVA using Bonferroni t-test to assess the significant differences.

## 3 Results

### 3.1 DON exposure evokes rapid and transient anorectic response as well as pro-inflammatory cytokine release

**Figure 2:**
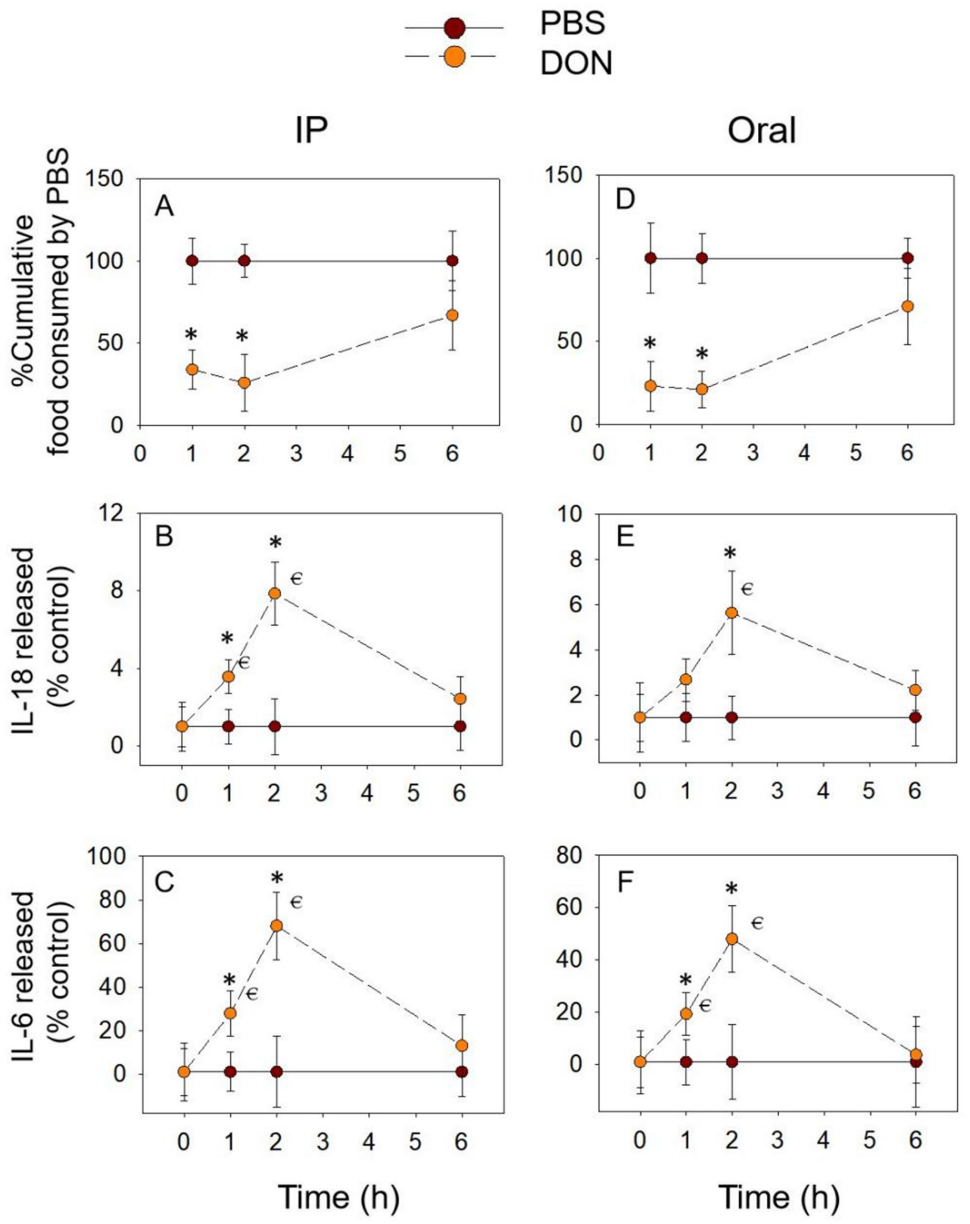
DON-induced anorexia and expression of IL-18 and IL-6. Mice were administered with PBS or DON (2.5 mg/kg BW). IP **(A)** and Oral **(D)** exposure to DON induced a rapid anorexia response in mice. Effect of DON on plasma IL-18 **(B,E)** and IL-6 **(C,F)** levels, % Cumulative food consumed by PBS = Cumulative food intake of mice administered with DON / Cumulative food intake of mice administered with PBS, IL-18 released (% control) = Plasma IL-18 in the mice administered with DON / Plasma IL-18 in the mice administered with PBS, IL-6 released (% control) = Plasma IL-6 in the mice administered with DON / Plasma IL-6 in the mice administered with PBS, the same as below. *p<0.05, compared to the control at a specific time point. *p*<0.05 compared to the control at 0 h.

Compared to the PBS group, either oral or IP administration of DON significantly reduced the cumulative food intake of the mice within 2 h (Figures 2A,D). At 6 h, the cumulative food intake of the DON-exposed group returned to normal levels. The expression of the pro-inflammatory cytokines IL-18 (Figures 2B,E) and IL-6 in the both groups showed the same pattern of gradual increase over 2 h, reaching a peak at 2 h (Figures 2C,F). No effect of DON exposure on IL-18 and IL-6 at 6 h. In summary, the temporal relationship between pro-inflammatory cytokines IL-18 and IL-6 and cumulative food intake showed an opposite trend, suggesting that IL-18 and IL-6 may be involved in the DON-induced food refusal response.

### 3.2 3-ADON exposure also evokes rapid and transient anorectic response as well as pro-inflammatory cytokine release

**Figure 3:**
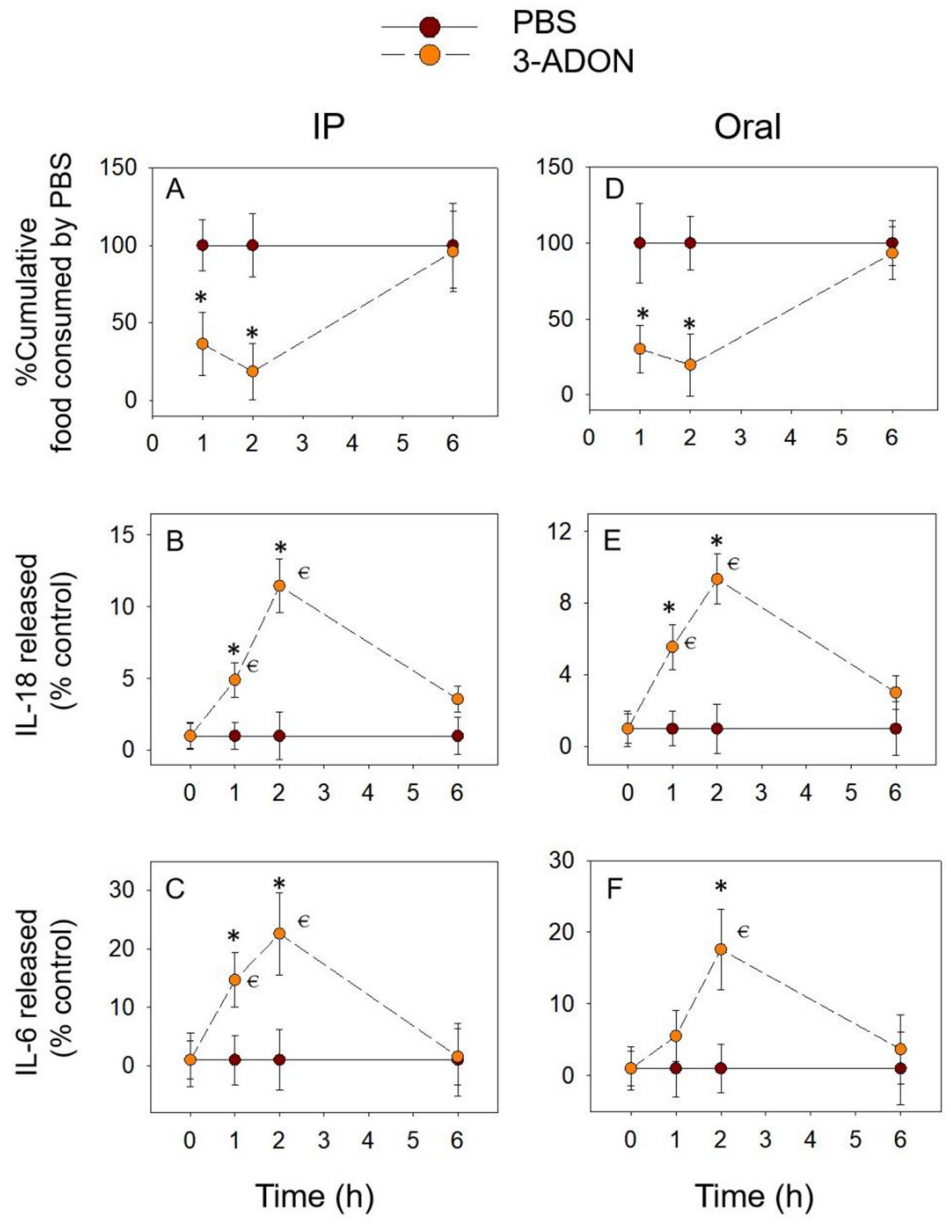
3-ADON-induced anorexia **(A,D)** corresponds to plasma IL-18 **(B,E)** and IL-6 **(C,F)** elevation. Mice were administered with either PBS or 3-ADON (2.5 mg/kg BW). *p<0.05, compared to the control at a specific time point. *p*<0.05 compared to the control at 0 h.

The temporal relationship of 3-ADON to cumulative food intake and pro-inflammatory cytokine expression followed the pattern of DON. 3-ADON cumulative food intake also decreased at 1 h and at 2 h and reached a nadir at 2 h, returning to normal levels at 6 h. IL-18 and IL-6 increased significantly at 2 h and gradually returned to basal levels at 6 h (*p* > 0.05). The above results express that the cumulative food intake due to 3-ADON shows an opposite trend concerning the temporal relationship between the pro-inflammatory cytokines IL-18 and IL-6, which may indicate that IL-18 and IL-6 are equally involved in 3-ADON-induced food refusal (Figure 3).

### 3.3 15-ADON exposure also causes rapid and transient anorectic response as well as pro-inflammatory cytokine release

**Figure 4:**
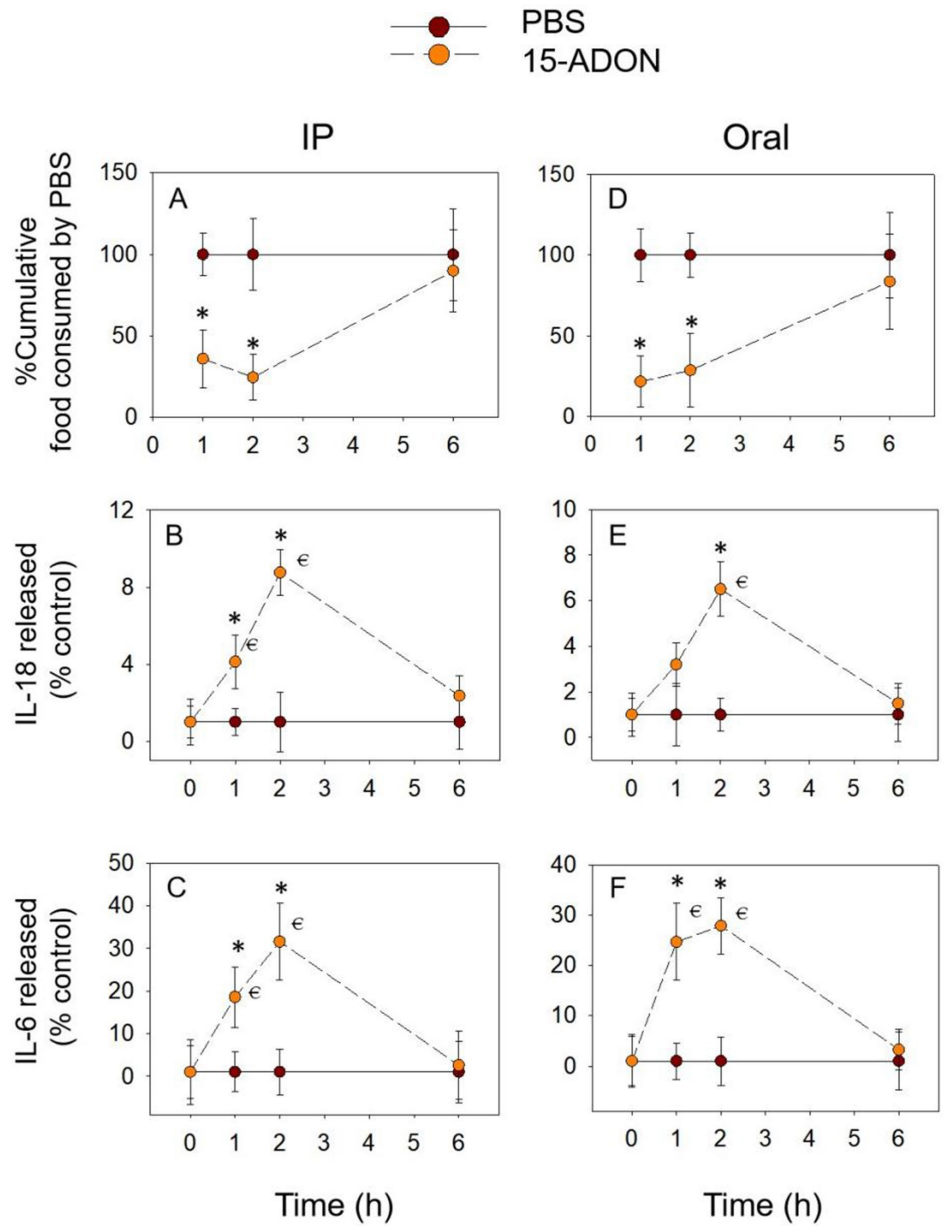
15-ADON-induced anorexia **(A,D)** corresponds to plasma IL-18 **(B,E)** and IL-6 **(C,F)** elevation. Mice were administered with either PBS or 15-ADON (2.5 mg/kg BW). *p<0.05, compared to the control at a specific time point. *p*<0.05 compared to the control at 0 h.

After 15-ADON was administered intraperitoneally, cumulative food intake was significantly reduced for within 2 h and reached a nadir at 2 h (Figure 4A). After oral exposure with 15-ADON, the cumulative food intake at 2 h, although also significantly lower than in the PBS group, tended to increase compared to 1 h (Figure 4D). Cumulative food intake returned to normal at 6 h for both oral and IP groups (*p* > 0.05). The release of IL-18 (Figures 4B,E) and IL-6 (Figures 4C,F) increased from 1 h, peaked at 2 h and returned to normal levels at 6 h (*p* > 0.05). These results are similar to those described above for DON and 3-ADON, suggesting that IL-18 and IL-6 may also be involved in food refusal due to 15-ADON.

### 3.4 FX exposure induces prolonged anorectic response and pro-inflammatory cytokine release

**Figure 5:**
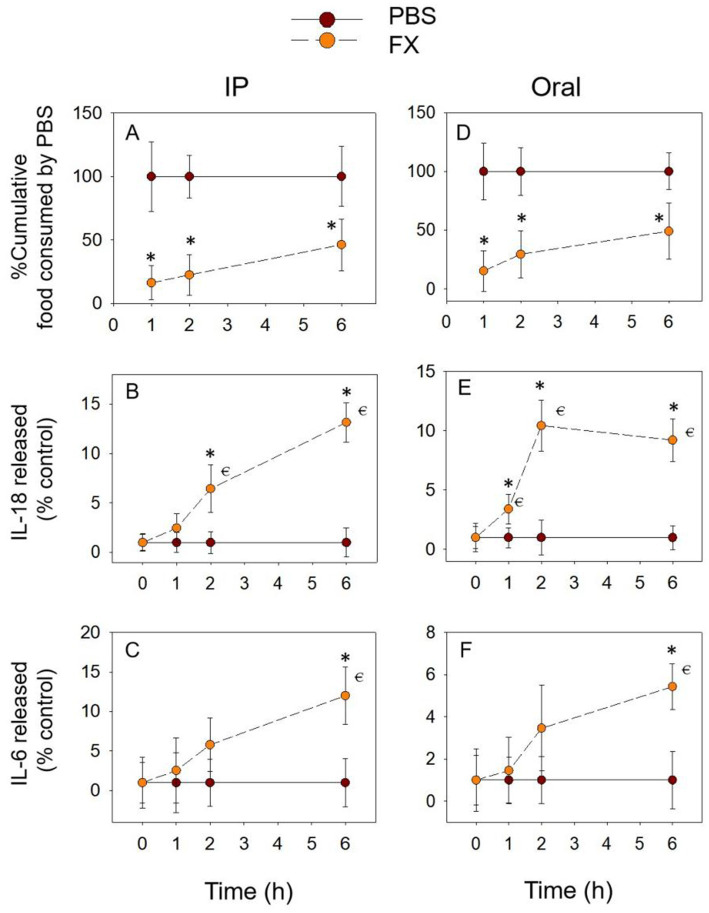
FX-induced anorexia **(A,D)** corresponds to plasma IL-18 **(B,E)** and IL-6 **(C,F)** elevation. Mice were administered with either PBS or FX (2.5 mg/kg BW). *p<0.05, compared to the control at a specific time point. *p*<0.05 compared to the control at 0 h.

As shown in Figures 5A,D, the FX-induced food refusal response may be stronger than the DON congeners described above. The cumulative food intake induced by FX at 6 h remained significantly lower than in the PBS group, whether exposure methods. With the IP group, IL-18 release gradually increased from 1 h, peaking at 6 h, and was significantly higher than in the PBS group at 2 h as well as at 6 h (Figure 5B). During gavage, IL-18 expression increased significantly at 1 h, 2 h, and 6 h, reaching a peak at 2 h (Figure 5E). The overall expression of IL-6 showed an increasing trend, reaching a peak and significance at 6 h (Figures 5C,F). In addition, the cumulative food intake decreased significantly at 6 h, while the expression of inflammatory cytokines increased significantly at this time. This also suggests a close relationship between the pro-inflammatory cytokines IL-18 and IL-6 and the food refusal effect caused by FX.

### 3.5 NIV exposure also induces prolonged anorectic response and pro-inflammatory cytokine release

**Figure 6:**
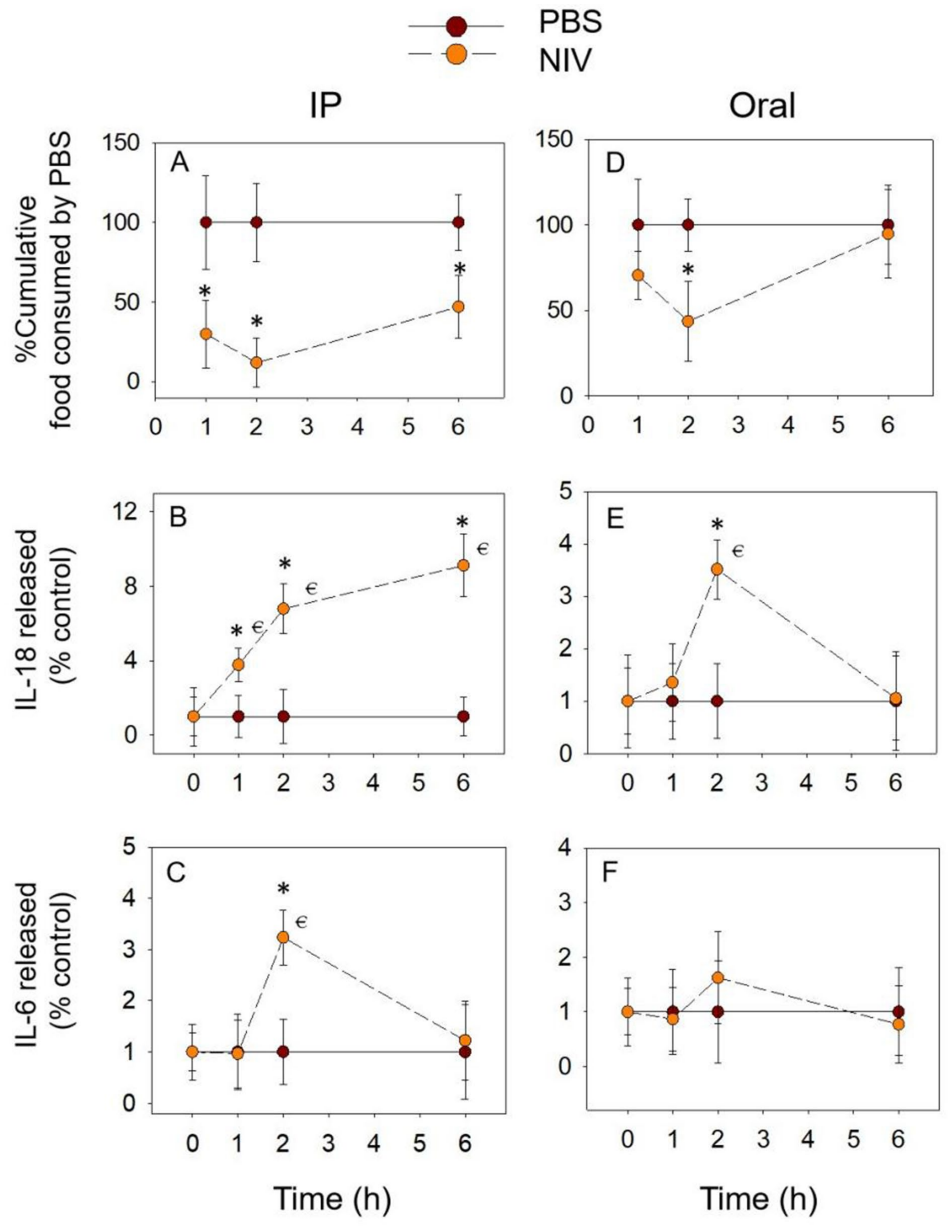
NIV-induced anorexia **(A,D)** corresponds to plasma IL-18 **(B,E)** and IL-6 **(C,F)** elevation. Mice were administered with either PBS or NIV (2.5 mg/kg BW). *p<0.05, compared to the control at a specific time point. *p*<0.05 compared to the control at 0 h.

The different exposure modes of NIV showed similar trends for cumulative food intake. The difference was that with IP group, the cumulative food intake was significantly reduced at 1 h. At 6 h, although it increased compared to 2 h, it was still significantly reduced compared to the PBS group (Figure 6A). With gavage exposure, only at 2 h was there a significant down-regulation of cumulative food intake (Figure 6D). With the IP group, IL-18 release gradually increased to a peak at 6 h and was significantly upregulated at all three-time points compared to the PBS group (Figure 6B). IL-18 release peaked at 2 h (Figure 6E) and returned to normal levels at 6 h (*p* > 0.05) when exposed by gavage. In comparison, IL-6 release was only significantly upregulated at 2 h when NIV was administered intraperitoneally and was not significantly different at all time points when oral exposure (*p* > 0.05) (Figures 6C,F). These data suggest that the effect of NIV-induced food refusal may be primarily related to IL-18.

## 4 Discussion

Mycotoxins are common contaminants in food, affecting approximately 25% of food crops and causing damage to nearly 100 million tonnes of food globally yearly (47). As one of the most common mycotoxins, DON has been widely reported for its toxicity and mechanism. In contrast to DON, the toxic effects of its congeners have rarely been reported. Several investigations of mycotoxin contamination in cereals have shown that contamination by DON congeners is also widespread (6–8, 48). Anorexia, as an important point in their toxicity, endangers the health of humans and animals. However, the mechanism by which they cause anorexia is not yet clear. In this study, we focused on two common pro-inflammatory cytokines (IL-18, IL-6), and investigated the anorexigenic potency of DON and its congeners and the release of IL-18 and IL-6 in plasma under different methods (oral vs. IP) based on anorexia models in mice. The data showed that exposure to DON and its congeners at 2.5 mg/kg BW significantly elevated plasma IL-18 and IL-6 levels associated with toxin-induced anorexia.

All five toxins significantly induced anorexia in mice within 2 h. Regardless of the exposure mode, the cumulative food intake of DON, 3-ADON, and 15-ADON had returned to normal levels in mice at 6 h (*p* > 0.05). The difference is that FX still induced food refusal at 6 h. NIV only returned to normal levels of cumulative food intake at 6 h when exposed orally. These results are similar to those previously reported (26, 49). In mice, the elimination half-lives of NIV, FX, and DON were 14.3 h, 37.6 h, and 11.8 h, respectively. This difference may help explain the significant anorexia induced by NIV and FX at 6 h (50). Poapolathep (5) reported that FX has higher oral toxicity than NIV in mice. Furthmore, the oral bioavailability of FX was higher than that of NIV in mice (51). It has been reported (52) that using small-molecule drugs by IP leads to faster and more complete absorption compared to oral exposure. This is similar to the previous results (53) that IP of these aforementioned mycotoxins induced a stronger anorexic than oral exposure.

IL-6 is a multifunctional inflammatory cytokine that functions in combination with the IL-6 receptor and glycoprotein 130 to form a hexameric complex. This complex can participate in the pathophysiological processes of the body by activating multiple signaling pathways (54). IL-18 belongs to the IL-1 cytokine family and is structurally related to IL-1β. The precursor of IL-18 is cleaved by caspase-1 to produce the active form, and upon binding to the corresponding receptor, IL-18 further causes a signaling cascade to activate NF-κB and MAPK signaling pathways (55, 56). DON promoted inflammatory cytokines such as IL-6, IL-1β, TNF-*α*, and IL-18 has been well reported (36, 42, 57). The data from this study also showed that both oral and IP of DON resulted in significant IL-18 as well as IL-6 expression, and this was an early process. In addition, 3-ADON and 15-ADON induced IL-6 expression similar to DON. Both exposure modes of FX significantly upregulated IL-6 at 6 h. NIV significantly upregulated IL-6 only after oral exposure at 2 h. Previously, the expression of inflammatory cytokines induced by DON congeners focused on IL-6, IL-1β, and TNF-*α* (37, 41). The present study may be the first systematic report of DON congeners-induced IL-18 expression. The effects of 3-ADON, as well as 15-ADON on IL-18, were similar to those of DON, with FX still significantly upregulating IL-18 expression at 6 h. Whereas NIV only had a significant difference at 6 h when injected intraperitoneally, when exposed orally, the IL-18 expression was significant only at 2 h.

In addition, the temporal relationship of DON and its congeners-induced IL-18, as well as IL-6 expression, was found to be consistent with their resulting anorexia. Inflammation has been reported to have a negative impact on the regulation of appetite and thus on food intake (44). Cytokines may reduce appetite by interacting with the hypothalamus thus leading to anorexia (58). Previous studies have shown that TNF-*α* and IL-1β play an important role in the induction of anorexia by DON and its congeners and that the use of antagonists of the TNF-α receptor as well as the IL-1β receptor also attenuates DON-induced anorexia (45, 49). DON crosses the blood–brain barrier and acts directly on the hypothalamus to induce the expression of the inflammatory cytokine (27, 59). Inflammatory cytokines IL-18 and IL-6 can further activate the NF-κB signaling pathway, regulating POMC activity to cause anorexia (27, 55, 60). In addition, it has been reported that inflammation-induced anorexia is due to elevated levels of circulating leptin. Pro-inflammatory cytokines can increase OB gene expression, leading to elevated plasma leptin levels (61, 62). In turn, leptin coordinates the control of food intake and energy expenditure (63). IL-6 may be involved in appetite regulation after acute exercise in humans (43). IL-6 produced during exercise may alter leptin in the ARC, affecting post-exercise eating behavior (64) IL-18 knockdown mice cause hyperphagia, obesity, and insulin resistance (65, 66). Insulin, in turn, can be involved in appetite regulation by regulating levels of neuropeptides (NPY and POMC) (67). IL-18 may inhibit feeding by suppressing the activity of bed nucleus of the stria terminalis (BST) type III GABAergic neurons (68).

There is often a complex interaction between inflammation and intestinal flora, with dysbiosis leading to intestinal inflammation and intestinal inflammation changing the intestinal microbiota (69). IL-22 has a dual role in the progression of inflammation, with IL-22-deficient mice altering the expression of antimicrobial peptides as well as microbial diversity in the colon (70, 71). IL-18 supplementation in NLRP6, ASC, and Caspase 1/11 knockout mice significantly alters the intestinal microbiota of mice (72). The relationship between intestinal flora and appetite has also been well reported, and intestinal microbial metabolites and components can act as appetite-related signaling molecules to regulate appetite-related hormone secretion or act directly on hypothalamic neurons (73). In addition, *E. coli* can produce an anorexigenic bacterial protein, caseinolytic protease B homolog protein (ClpB), which acts as an antigenic mimic of *α*-melanocyte-stimulating hormone (α-MSH) to trigger the production of α-MSH across reactive autoantibodies, which in turn can bind to MC4R and play an important role in satiety (74). In turn, exposure to DON disrupts the intestinal microbial structure of mice leading to ecological dysregulation (75, 76). Based on this, we hypothesized that the expression of inflammatory cytokines (IL-18, IL-6) induced by DON and its congeners may lead to anorexia by altering the composition of the intestinal flora and consequently. However, this still needs to be explored in further experiments. Moreover, we speculate that IL-18 and IL-6 in the toxin-induced anorexia response directly acts on the hypothalamus to regulate the appetite center. On the other hand, may indirectly regulate appetite through some key molecules such as leptin, eventually leading to anorexia. Moreover, intestinal flora may also play a role. However, these require further research to verify.

## 5 Conclusion

In summary, this study shows that both IP and oral treatment of DON and its four congeners 3-ADON, 15-ADON, FX, NIV significantly upregulated IL-18 and IL-6 expression in mouse plasma. Moreover, the temporal relationship between the expression of IL-18 and IL-6 was consistent with the anorexia caused by these mycotoxins. Future research should focus on exploring how these pro-inflammatory cytokines regulate appetite as well as the interaction between pro-inflammatory cytokines and brain-gut peptides such as GLP-1, CCK and SP. Especially, the potential roles of calcium sensing receptor (CaSR), transient receptor potential ankyrin 1 (TRPA1), transient receptor potential channel M5 (TRPM5) and transient receptor potential vanilloid subtype 1 (TRPV1) in the regulation of pro-inflammatory cytokines release. From veterinary and public health perspectives, studies such as this will improve our understanding the adverse effects of mycotoxins and provide a theoretical basis for the prevention and treatment of DON and its congeners poisoning.

## Data Availability

The original contributions presented in the study are included in the article/supplementary material, further inquiries can be directed to the corresponding authors.
